# Nuclear domain ‘knock-in’ screen for the evaluation and identification of small molecule enhancers of CRISPR-based genome editing

**DOI:** 10.1093/nar/gkv993

**Published:** 2015-10-01

**Authors:** Jordan Pinder, Jayme Salsman, Graham Dellaire

**Affiliations:** 1 Department of Pathology, Dalhousie University, Halifax, Nova Scotia, B3H 4R2, Canada; 2 Department of Biochemistry & Molecular Biology, Dalhousie University, Halifax, Nova Scotia, B3H 4R2, Canada; 3 Beatrice Hunter Cancer Research Institute, Halifax, Nova Scotia, B3H 4R2, Canada

## Abstract

CRISPR is a genome-editing platform that makes use of the bacterially-derived endonuclease Cas9 to introduce DNA double-strand breaks at precise locations in the genome using complementary guide RNAs. We developed a nuclear domain knock-in screen, whereby the insertion of a gene encoding the green fluorescent protein variant Clover is inserted by Cas9-mediated homology directed repair (HDR) within the first exon of genes that are required for the structural integrity of subnuclear domains such as the nuclear lamina and promyelocytic leukemia nuclear bodies (PML NBs). Using this approach, we compared strategies for enhancing CRISPR-mediated HDR, focusing on known genes and small molecules that impact non-homologous end joining (NHEJ) and homologous recombination (HR). Ultimately, we identified the small molecule RS-1 as a potent enhancer of CRISPR-based genome editing, enhancing HDR 3- to 6-fold depending on the locus and transfection method. We also characterized U2OS human osteosarcoma cells expressing Clover-tagged PML and demonstrate that this strategy generates cell lines with PML NBs that are structurally and functionally similar to bodies in the parental cell line. Thus, the nuclear domain knock-in screen that we describe provides a simple means of rapidly evaluating methods and small molecules that have the potential to enhance Cas9-mediated HDR.

## INTRODUCTION

The recent development of systems for creating site-specific DNA double-strand breaks (DSBs) has enabled precise engineering of the mammalian genome. Several classes of endonucleases have been reengineered for induction of targeted DSBs in mammalian cells, including transcription activator-like effector nucleases (TALENs), zinc finger nucleases (ZFNs) and more recently, the *Streptococcus pyogenes* clustered regularly interspaced short palindromic repeats (CRISPR)/Cas9 machinery (1–3).

The popularity of the CRISPR/Cas9 system has surpassed both ZFNs and TALENs in part due to the ease of modulating target specificity and the wide availability of reagents for research use through the plasmid repository Addgene (www.addgene.org). The Cas9 endonuclease is directed to a locus through binding of a single guide RNA (gRNA) to its complementary genomic DNA target. Specificity is achieved by modifying the 20-nt region of the gRNA that recognizes the sequence next to a trinucleotide (NGG) protospacer adjacent motif (PAM) (4–6). To minimize potential off-target cleavage caused by association of a gRNA with multiple sites in the genome, the Cas9^D10A^ nickase mutant can be used, which creates single-strand breaks (SSBs) instead of DSBs (7). When the Cas9^D10A^ nickase is expressed with two gRNAs targeting opposite strands within close proximity, a DSB will be created, while other regions targeted by each individual gRNA will only incur single-strand nicks, which are efficiently and faithfully repaired (7).

Repair of Cas9-induced DSBs by error-prone non-homologous end joining (NHEJ), the predominant DSB break repair pathway in mammalian cells, can be exploited to generate a knockout phenotype. Repair of a single DSB creates a small deletion, while multiple DSBs within a region can generate large deletions, from a few kilobases to megabases (7–9). Alternatively, the DSB can be repaired by homology directed repair (HDR). To insert or replace a DNA sequence near the break site, a DNA fragment to be used as a template for repair is introduced. The repair template contains homology to the regions flanking the DSB; repair of the break leads to insertion of the repair template without introducing extraneous bases (3). Thus via HDR, scarless insertion of DNA into the mammalian genome can be used to create precise deletions, base substitutions, or insertion of coding sequences for epitope tags, such as fluorescent proteins. This technology has also been applied to engineering genomes of other eukaryotes, such as yeast, flies and zebrafish (10–13). Studying the effects of mutation or depletion of a protein in a cell line in which a gene is modified at its endogenous locus is preferable over the current standard methods. Knockdown of endogenous gene expression with short-hairpin RNA (shRNA) or interfering RNA can be effective; however, these approaches vary in their knockdown efficiency. Additionally, the risk of off-target effects of the hairpin can mitigate the benefits or confound the interpretation of these strategies (14–16). If the target RNA is part of a microRNA regulatory network, silencing the transcript with shRNA could disrupt the entire network (17). For expression studies, genes of interest are often expressed ectopically from a plasmid or by using retroviruses that integrate the transgene randomly in the genome. Modern genome editing techniques like CRISPR/Cas9 can now be used to preserve a gene's endogenous promoter and transcriptional and post-transcriptional regulation, including microRNA regulation and alternative splicing, thus alleviating some of the complicating factors inherent to other methods.

One current limitation of Cas9-induced HDR is low efficiency. Even with a high transfection efficiency of expression vectors encoding Cas9, the gRNA and the repair template, only a small proportion (<10%) of cells undergo the recombination event (3,18,19). In contrast, the efficiency of creating deletions by NHEJ approaches 90% (19,20). This may reflect higher utilization of the NHEJ pathway over homologous recombination (HR) in mammalian cells. Inhibiting NHEJ has been shown to increase rates of HR (21,22). Optimizing the efficiency of HDR would facilitate the rapid generation of cell lines with precisely edited genes but also the ability to generate genetically modified clonal cell lines even in the absence of a selectable phenotype, which may prove useful in the application of Cas9 technology to gene therapy.

In this study, we have used a flow cytometry-based assay to optimize CRISPR/Cas9-mediated knock-in events by HDR, employing a number of strategies. Ultimately, we show that small-molecule activation of RAD51, a protein involved in strand exchange and the search for homology during HDR (23), enhances the efficiency of Cas9-stimulated HDR by up to 3-fold. We apply our findings to generate a cell line to be used for live cell studies of promyelocytic leukemia nuclear bodies (PML NBs). Previous live cell studies using green fluorescent protein (GFP)-tagged PML NBs relied on over-expression of a single PML isoform, resulting in larger, more numerous PML NBs (24,25) that lack the normal heterogeneous constitution of the six nuclear PML isoforms (26–28). We show that insertion of the coding sequence for the green fluorescent protein Clover after the second codon of PML leads to multiple isoforms of PML being expressed as a Clover fusion. Clover-labeled PML bodies co-localize with known PML NB proteins and, like normal PML NBs, increase in number in response to interferon. The cell line validates the utility of CRISPR/Cas9-mediated gene targeting and is a novel tool for live cell studies of PML dynamics.

## MATERIALS AND METHODS

### Cell culture

HEK-293A human embryonic kidney and U2OS osteosarcoma cell lines (parental U2OS and U2OS^Clover–PML^) were cultured in Dulbecco's modified Eagle's medium (Life Technologies) supplemented with 10% fetal calf serum, at 37°C with 5% CO_2_. U2OS cell lines stably expressing GFP-PML-I and GFP-PML-IV (29) were maintained as above with the addition of 800 (PML-I) or 1600 (PML-IV) μg/ml G418. The RAD51-stimulatory compound RS-1 (Sigma), the DNA ligase IV inhibitor SCR7 (1 μM, XcessBio) and HDR stimulatory compound L755507 (5 μM, Tocris) were added to culture media from 10 mg/ml stock solutions in DMSO.

### Alamar blue assay

For measuring the effect of RS-1 on metabolic activity by reduction of Alamar blue, cells were grown for three days in the presence or absence of RS-1, and fresh media containing 10% Alamar blue was added. Absorbance was measured 4 h later at a wavelength of 562 nm and 595 nm.

### Plasmid construction

Plasmids used in this study are described in Supplementary Table 1. For expression of chimeric gRNAs targeting various protospacer sequences in the genome, oligonucleotides were annealed and cloned into BbsI-digested pX330-U6-Chimeric_BB-CBh-hSpCas9 (Addgene plasmid #42230), for editing the *LMNA* locus, or pX335-U6-Chimeric_BB-CBh-hSpCas9n(D10A) (Addgene plasmid # 42335), for editing the *PML* locus, which were gifts from Feng Zhang (2). The gRNA sequences were analyzed using the COSMID (CRISPR Off-target Sites with Mismatches, Insertions and Deletions) website (http://crispr.bme.gatech.edu/) to check the sgRNA against the GRCh38 (hg38) genome build for potential off-target sites (30).

pcDNA3-Clover was a gift from Michael Lin (Addgene plasmid # 40259) (31). piRFP670-N1 was a gift from Vladislav Verkhusha (Addgene plasmid # 45457) (32). Homology repair templates were generated by PCR using Phusion high-fidelity polymerase (New England BioLabs) and cloned into pCR2.1 using a TOPO-TA Cloning Kit (Life Technologies). Site-directed mutagenesis of the BRCA1 in pHA-BRCA1 (a gift from David Livingston) (33) was carried out using overlap extension PCR.

### Transfections

For lipofection, cells were transfected with DNA using Lipofectamine 2000 (Life Technologies) according to the manufacturer's instructions. For electroporation, cells were transfected using the Neon transfection system (Life Technologies) using 100 μl pipette tips with the manufacturer's recommending settings.

### Flow cytometry

For determining the percent of Clover-positive cells, cells were washed in PBS, fixed in 2% paraformaldehyde for ≥20 min and analyzed by flow cytometry using either a FACS Aria III cell sorter (BD Biosciences) or LSR-Fortessa (BD Biosciences). Cells were first gated for the intact cell population using forward scatter versus side scatter plots, and then gated for transfected cells based on the presence of the transfection control, far red protein iRFP670, using the side scatter versus iRFP670 plots. Transfected cells were gated for Clover-positive cells on the side scatter versus Clover plots such that the percentage of untransfected cells designated as Clover-positive was between 0–1%. The means and standard errors from at least three independent experiments were calculated and statistical significance was determined using the student's t-test.

### Genomic DNA extraction and PCR validation of U2OS^Clover–PML^ cells

Genomic DNA was extracted from U2OS cell pellets using the Wizard Genome DNA Purification Kit (Promega, A1125) as per the manufacturer's instructions and amplified using primers specific to the *PML* gene locus surrounding exon 1 (PML forward primer: 5′- CTACCTCTCCCGCTTTACCG -3′, PML reverse primer 5′- TGTCTGGACTTCCCGCTTTG -3′). PCR amplicons were gel purified and sequenced (Genewiz Inc.).

### Surveyor assay

Surveyor assays were performed using a Surveyor Mutation Detection Kit (Integrated DNA Technologies) according to the manufacturer's directions. Indel percentage was determined by the formula 100 × (1 −(1 − *b*) / (*a* + *b*))^1/2^ where *a* is the integrated intensity of the undigested PCR product and *b* is the combined integrated intensity of the cleavage products (7).

### Western blotting and immunofluorescence microscopy

Antibodies for western blotting and immunofluorescence analysis were sheep anti-PML (PML2A, Diagnostics Scotland), rabbit anti-PML (Bethyl Laboratories, A301–167A), mouse anti-PML (E-11, Santa Cruz Biotechnology, sc377390), rabbit anti-Sp100 (Chemicon, ab1380), rabbit anti-Daxx (Sigma, D7810), chicken anti-GFP (Abcam, ab13970), mouse HRP-conjugated anti-GAPDH (abcam ab9482).

For western blot analysis, cells were recovered from 10 cm culture dishes, washed with PBS and lysed in RIPA buffer (Sigma) with protease inhibitors (P8340, Sigma) for 20 min on ice. Lysates were cleared (10 min, 15 000 × g, 4°C) and protein extracts were analyzed by SDS-PAGE and western blotting using 5% milk powder 0.1% Tween 20 in PBS as a blocking solution.

U2OS and U2OS^Clover–PML^ cells were plated onto coverslips in a 6-well plate and treated with 1000 IU IFNγ (R&D systems, cat# 285-IF) for 24 h. Cells were washed with PBS and fixed in 4% paraformaldehyde for 10 min. Cells were permeabilized with 0.5% Triton X-100 in PBS and blocked with 4% BSA in PBS. Cells were then immunolabeled with primary antibodies (1 h, diluted in blocking buffer), washed with PBS and incubated with fluorescently labeled secondary antibodies (45 min, diluted in blocking buffer). Cells were washed several times in PBS and incubated with 1 μg/m^l^ of 4′,6-diamidino-2-phenylindole (DAPI) stain to visualize DNA. Fluorescent images were captured with a HQ2 CCD camera (Photometrics) on a Zeiss Cell Observer Microscope using a 63X (1.4 NA) immersion oil objective lens. Images were processed using only linear adjustments (e.g. brightness/contrast) with Slidebook (Intelligent Imaging Innovations, Boulder, CO) and Adobe Photoshop CS5. Images were individually adjusted to provide sufficient contrast to best visualize nuclear bodies (e.g. PML, Sp100, Daxx) for colocalization analysis and for accurate quantification of PML nuclear body number. Where appropriate (i.e. Figure 7), representative images were selected to best reflect the quantification data.

Quantification of PML NBs was determined by counting the number of PML NBs in each of 50 cells per experimental condition and calculating the average number of PML bodies per cell. The means and standard errors from at least three independent experiments were calculated and statistical significance was determined using the student's t-test in Microsoft Excel 2007.

## RESULTS

### A flow cytometry assay for measuring efficiency of Cas9-induced HDR

**Figure 1.**
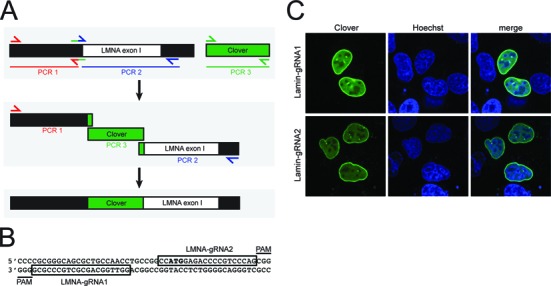
Cas9-directed knock-in of Clover in the LMNA coding sequence. (**A**) The repair template sequence was created by overlap extension PCR from three PCR products, containing the 5′ homology arm (PCR1), 3′ homology arm (PCR2) and the Clover coding sequence (PCR3). Primers are listed in Supplementary Table 1. The products were gel-purified and used as templates in a second PCR reaction using primers that anneal to the distal ends of the homology arms. This product was TA-cloned to create pCR2.1-CloverLamin. (**B**) Two 20-nt gRNA target sites were selected near the LMNA start codon (bold). (**C**) U2OS cells were co-transfected with plasmids encoding the pCR2.1-CloverLamin, and a plasmid pX330-LMNA1 or pX330-LMNA2 which encode Cas9 and one of the two chimeric gRNAs (2) targeting either the LMNA-gRNA1 or LMNA-gRNA2 sequence. Three days later cells were stained with 2 μM Hoechst 33342 and imaged by confocal microscopy.

To develop an assay for measuring Cas9-induced knock-in efficiency, a flow cytometry assay was designed to assess the percentage of cells that have undergone HDR. CRISPR reagents were designed to insert the sequence for the monomeric green fluorescent protein Clover, which is 2.5 times brighter than EGFP (31), after the second codon of the *LMNA* gene, which encodes the lamin A and lamin C isoforms. Using these reagents we intended to generate cells expressing fluorescent nuclear lamin A/C as a means of marking cells that had undergone productive HDR. Lamins display a distinct localization pattern and are expressed in virtually all cell types (34). Homology arms corresponding to regions flanking the *LMNA* start codon were amplified by PCR from genomic DNA isolated from U2OS osteosarcoma cells, and were cloned into a vector flanking the Clover coding sequence (Figure 1A). Two 20-nt gRNA target sites were selected within the genomic region near the *LMNA* start codon (Figure 1B, Supplementary Table 2). U2OS cells were co-transfected with the Clover homology repair template plasmid and a plasmid expressing Cas9 and a chimeric gRNA containing either of the two selected 20-nt protospacer sequences (2). For each gRNA, a subpopulation of cells exhibited a pattern of green fluorescence enriched at the nuclear periphery as expected for Clover-labeled lamins (Figure 1C), indicating that the Clover sequence had been successfully inserted in-frame into the *LMNA* gene.

### The RAD51 agonist RS-1 improves efficiency of Cas9-induced HDR

**Figure 2.**
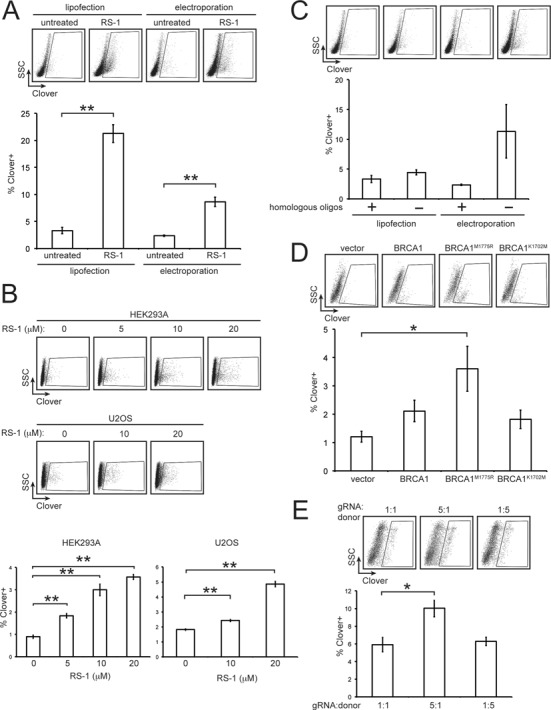
Testing strategies for enhancing Cas9-mediated HDR. HEK293A cells at 90% confluence in 12-well plates were co-transfected by lipofection with a pX330-LMNA1, and pCR2.1-CloverLamin at a 1:1 ratio of 0.5 μg of each. In addition, 0.1 μg piRFP670-N1 was included per transfection as a transfection control. Three to five days later cells were fixed and analyzed by flow cytometry. Shown are representative side scatter versus Clover plots and the mean percentage of Clover-positive cells from three replicate experiments (± S.E.M.) counting at least 10 000 events per experiment. Student's t-test results are indicated with asterisks (* = *P* < 0.05, ** = *P* < 0.01). (**A**) Cells were transfected by lipofection or electroporation and grown in the presence or absence of 10 μM RS-1. (**B**) HEK293A or U2OS cells were transfected with the same plasmids in (**A**) and grown in the presence of the indicated concentrations of RS-1. (**C**) Cells were transfected by lipofection or electroporation and were supplemented with 0.5 μg of oligonucleotides homologous to the ends of the repair template homology arms. (**D**) The amounts of the donor and gRNA plasmids were halved (0.25 μg of each) and 0.5 μg of a plasmid encoding HA-tagged BRCA1, the BRCA1 hyper-recombination mutants BRCA1^M1775R^ or BRCA1K^1702M^, or vector with no insert was added. (**E**) Cells were transfected with the indicated ratio (by mass) of pX330-LMNA1 to pCR2.1-CloverLamin, totalling 2 μg of DNA. Amounts were 0.5 μg of each plasmid, 1.6 μg of pX330-LMNA1 and 0.4 μg pCR2.1-CloverLamin, and 0.4 μg pX330-LMNA1 and 1.6 μg of pCR2.1-CloverLamin for the 1:1, 5:1 and 1:5 ratios, respectively.

NHEJ and HDR are the two major double strand break repair pathways in eukaryotes, with NHEJ being the predominant pathway in mammalian cells. We hypothesized that increasing the activity of proteins involved in HR may promote Cas9-induced HDR. We tested whether activation of RAD51 affects HDR. RAD51 is a nucleofilament-forming protein involved in the search for homology and strand invasion during HR repair. The compound RS-1 was identified in a screen for molecules affecting RAD51 activity, and was shown to stabilize association of RAD51 with DNA (35). To test whether RS-1 affected the efficiency of Cas9-stimulated HDR, HEK293A cells were co-transfected by either lipofection or electroporation with CRISPR plasmids for generating Clover-tagged lamins, grown in the presence or absence of RS-1 and analyzed by flow cytometry. RS-1 significantly improved the percent of Clover-positive cells for lipofection and electroporation transfection methods by ∼6-fold (*P* < 0.01) and ∼3-fold (*P* < 0.05), respectively (Figure 2A). This suggests that supplementing culture media with RS-1 may be a simple means of enhancing Cas9-stimulated gene targeting.

In gene targeting experiments using CRISPR/Cas9, repair of Cas9-induced DNA double-strand breaks can occurs primarily by either NHEJ or HR. While RS-1 enhanced HDR efficiency at the *LMNA* locus in HEK293A cells, it is possible that NHEJ events at the locus also increased. NHEJ is error-prone and an increase in NHEJ by RS-1 could lead to increased frequency of off-target mutations. To test whether RS-1 affects the frequency of error-prone repair of Cas9-induced DNA DSBs, HEK293A cells transfected with the gRNA and HDR constructs for Clover-tagging the *LMNA* gene. Three days following transfection genomic DNA was harvested and PCR products were amplified from either the LMNA locus or several predicted off-target loci and subjected to a Surveyor nuclease assay. We could not detect cutting and indel events by Surveyor assay at the predicted off-target loci we tested, which prevented further analysis of the effects of RS-1 at these loci. For the on-target locus, we observed no difference in the percentage of indel mutations between cells treated or untreated with RS-1 at the *LMNA* locus by Surveyor assay (Supplementary Figure S1). In addition, the Surveyor assay did not detect indels in cells treated with or without RS-1 in the absence of the Cas9 nuclease (data not shown).

To optimize the concentration of RS-1 for HDR enhancement, we examined the cytotoxicity (Supplementary Figure S2) and HDR enhancement (Figure 2B) of increasing concentrations of RS-1 in both HEK293A and U2OS cells. Alamar blue assays demonstrated that RS-1 exhibited cytotoxicity at concentrations of ≥10 μM and ≥1 μM for HEK293A and U2OS cells treated for 72 h, respectively (Supplementary Figure S2). Treatment with 10 μM RS-1 was only mildly cytotoxic and significantly enhanced HDR for both cell lines (Figure 2B); therefore, 10 μM RS-1 was used for subsequent experiments.

### Effect of homologous oligonucleotides and BRCA1 over-expression on CRISPR HDR efficiency

Single stranded oligonucleotides homologous to the ends of a sequence to be inserted have been shown to enhance the efficiency of gene targeting in *Dictyostelium discoideum* (36). To test whether homologous oligonucleotides improve Cas9 gene targeting, 20 bp single-stranded DNA oligonucleotides homologous to the ends of the Clover-Lamin homology donor sequence were co-transfected into HEK293A cells along with the CRISPR constructs for generating Clover-tagged Lamins. We found no significant change in the percentage of Clover-positive cells when the oligonucleotides were included (Figure 2C).

BRCA1 is an E3 ubiquitin ligase that has an essential role in HR repair and is a key protein in influencing DNA repair pathway choice (37,38). Cells expressing BRCA1 variants containing amino acid substitutions in the BRCA1 C-terminus (BRCT) domain display a hyper-recombination phenotype, exhibiting increased rates of HR (39). To test whether transient over-expression of wild-type BRCA1 or hyper-recombination alleles affect CRISPR HDR efficiency, the HDR assay was carried out in cells that were additionally transfected with a plasmid encoding wild-type BRCA1, or the variants BRCA1^K1702M^ or BRCA1^M1775R^. Cells over-expressing wild-type BRCA1 and either the BRCA1^K1702M^ and BRCA1^M1775R^ hyper-recombination variants appeared to exhibit increased rates of HDR; however, only over-expression of BRCA1^M1775R^ led to a statistically significant increase in Clover-positive cells (*P* = 0.04) (Figure 2D).

### Increasing the ratio of gRNA/Cas9 plasmid to homology repair template improves HDR efficiency

Since multiple DNA molecules are co-transfected into cells to perform CRISPR-mediated HDR, an optimal ratio of the gRNA/Cas9 plasmid to the homology repair template plasmid may need to be determined to maximize gene targeting efficiency. To test this, HDR efficiency was assessed in cells transfected with either a 1:1, 5:1 or 1:5 ratio of Cas9/gRNA plasmid to homology repair template plasmid. We found that a 5:1 ratio led to an increase in gene targeting over a 1:1 ratio (*P* = 0.03), while decreasing the level of the repair template by 5-fold had no significant effect on HDR efficiency (Figure 2E). This suggests that for this assay, increasing the levels of Cas9 and chimeric gRNAs improves gene targeting.

### Assessing combined effects of RS-1 with the HDR-enhancing molecules SCR7 and L755507

**Figure 3.**
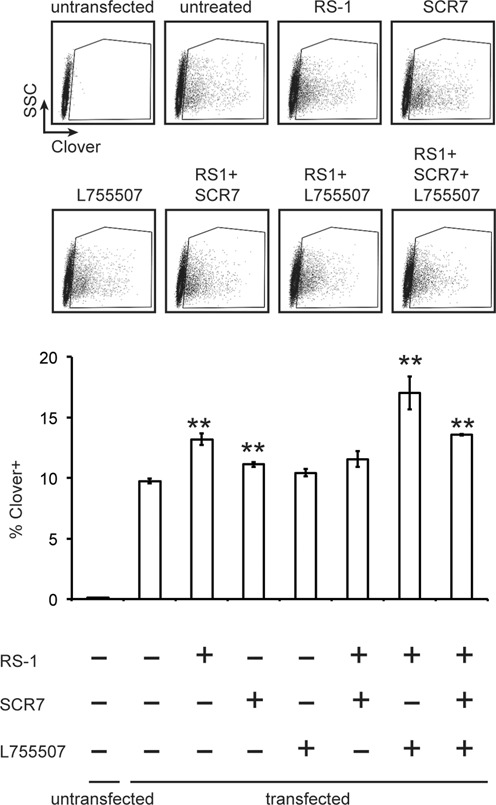
Testing HDR enhancement by RS-1, SCR7 and L755507. HEK293A were co-transfected by lipofection with 1 μg pX330-LMNA1, and 1 μg pCR2.1-CloverLamin. In addition, 0.1 μg piRFP670-N1 was included per transfection as a transfection control. Cells were cultured in the absence of drug or in the presence of 10 μM RS-1, 1 μM SCR7 or 5 μM L755507 as indicated. Three days following transfection cells were fixed and analyzed by flow cytometry. Shown are representative side scatter (SSC) versus Clover plots and the mean percentage of Clover-positive cells from three replicate experiments (±S.E.M.) counting at least 10 000 events per experiment (** = *P* < 0.01).

Inhibiting NHEJ by depleting the NHEJ pathway proteins such as Ku70 and XRCC4 has been shown to promote HDR (21). SCR7 is a small molecular inhibitor of the early NHEJ pathway protein DNA ligase IV (40) that has been shown to enhance HDR using CRISPR/Cas9 (22,41). Another study identified the molecule L755507 in screen for molecules that enhance HDR using CRISPR. To test whether RS-1 enhances the effects of SCR7 and L755507 on Cas9-induced HDR, HEK293A cells were transfected using the optimized 5:1 Cas9/gRNA plasmid to homology repair template plasmid ratio and cultured in media in the presence or absence of RS-1, SCR7 and L755507 (Figure 3). Both RS-1 (*P* = 0.003) and SCR7 (*P* = 0.009) independently enhanced HDR. Treatment with L755507 appeared to slightly stimulate HDR; however, the difference was not statistically significant (*P* = 0.12). Although combining RS-1 with L755507 appeared to partially synergize to increase HDR frequency, the difference over treatment with RS-1 alone was not significant (*P* = 0.06). Similarly, SCR7 in combination with RS-1 (or indeed treatment with L755507 combined with SCR7 and RS-1) failed to increase HDR over that seen with RS-1 alone. To control for the possibility of increased fluorescence due to the drugs, untransfected cells were treated with each combination of drugs and analyzed using identical flow cytometry parameters (Supplementary Figure S3). Thus, these results suggest that single agent treatment with RS-1 is potentially superior to treatment with L755507 or SCR7, and combining these compounds provides little additional benefit for the enhancement of HDR in our knock-in system.

### Using CRISPR/Cas9 HDR to generate fluorescently labeled PML NBs

**Figure 4.**
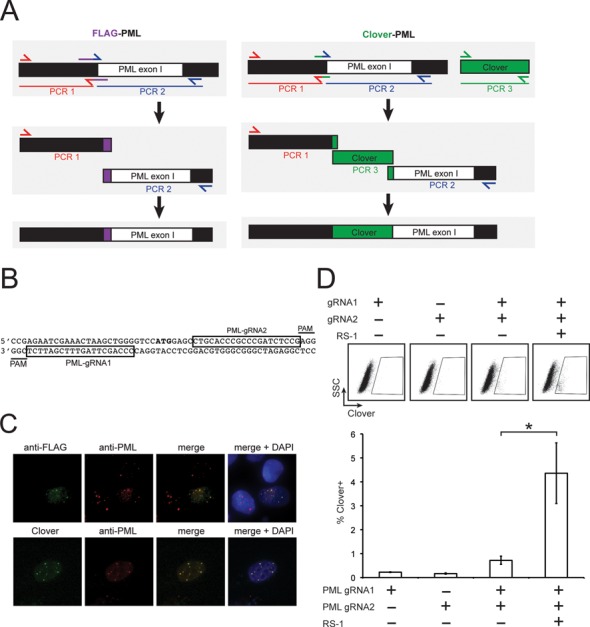
Generating cell lines expressing tagged PML protein. (**A**) Repair template sequences designed to insert either a FLAG or Clover epitope in-frame after the second codon of the *PML* gene were created by overlap extension PCR as described in Figure 1A and TA-cloned to make pCR2.1-FLAGPML and pCR2.1-CloverPML. Primers are listed in Supplementary Table 1. (**B**) Two gRNA target sites near the PML start codon (bold) were selected such that a DSB is created when simultaneously targeted by the Cas9^D10A^ nickase. (**C**) U2OS cells were transfected with a plasmid expressing wild-type Cas9 and chimeric PML-gRNA1, and either pCR2.1-FLAGPML or pCR.1-CloverPML. Three days after transfection cell were analyzed by immunofluorescence with the indicated antibodies and stained with DAPI. (**D**) HEK293A cells were transfected with pCR2.1-CloverPML, and plasmids expressing the Cas9^D10A^ nickase, and chimeric PML-gRNA1 and PML-gRNA2. 20 h post transfection, 10 μM RS-1 was added. Two days later, cells were fixed and the percent of Clover-positive cells was determined by flow cytometry (* = *P* < 0.05).

Tagging a gene at its endogenous locus is particularly advantageous when it is critical to maintain the normal expression level of the tagged protein. For a gene that encodes multiple splice isoforms, inserting the tag in a common exon can ensure that all isoforms contain the tag. The *PML* gene is an excellent candidate for tagging by CRISPR HDR since it encodes seven major isoforms that share a common N-terminus and vary in their C-termini (42). Previous studies on live cell PML NB dynamics examined cells encoding a single GFP-tagged isoform expressed under the control of a strong, constitutive promoter (24,25,29). These cells exhibit highly elevated levels of PML, increased PML NB number and altered isoform heterogeneity within PML NBs. To improve upon this model for studies of PML NB dynamics, constructs were designed to generate cell lines in which either a small (FLAG) or large (Clover) epitope was fused to the N-terminus of PML. Homology arms were amplified from U2OS genomic DNA and flanked either a FLAG epitope or Clover coding sequence for insertion in-frame immediately downstream of the second codon in PML (Figure 4A). Two gRNA target sequences were selected near the PML start codon (Figure 4B). To test whether the FLAG- and Clover-tagged PML localized to PML NBs, U2OS cells were co-transfected with plasmids expressing Cas9 and one of the chimeric PML gRNAs, and the homology donor plasmids for FLAG- or Clover-tagging PML. Immunofluorescence analyses showed that a subpopulation of cells transfected with the FLAG and Clover homology donor plasmids displayed anti-FLAG and Clover staining patterns, respectively, that co-localized with anti-PML staining in PML NBs, suggesting that in those cells the tags were effectively inserted in-frame and that N-terminally tagged PML could form PML NBs (Figure 4C).

To create a clonal cell line expressing Clover-tagged PML for live cell studies of PML, a double-nicking strategy was employed to minimize potential off-target effects of Cas9. For this method, two chimeric gRNAs targeting the *PML* gene are expressed with the Cas9^D10A^ nickase (7). To test whether RS-1 enhanced HDR at the *PML* locus using the double nicking technique, HEK293A cells were co-transfected with plasmids expressing the two chimeric *PML* gRNAs and the Cas9^D10A^ nickase, and the Clover–PML homology donor plasmid, and grown in the presence or absence of RS-1. RS-1 led to a ∼4-fold increase in the percent of Clover-positive cells (*P* = 0.04), suggesting that boosting the efficiency of Cas9-mediated HDR with RS-1 is compatible with the double-nicking technique for gene targeting (Figure 4D).

### Validation and characterization of a U2OS cell line expressing Clover-tagged PML

**Figure 5.**
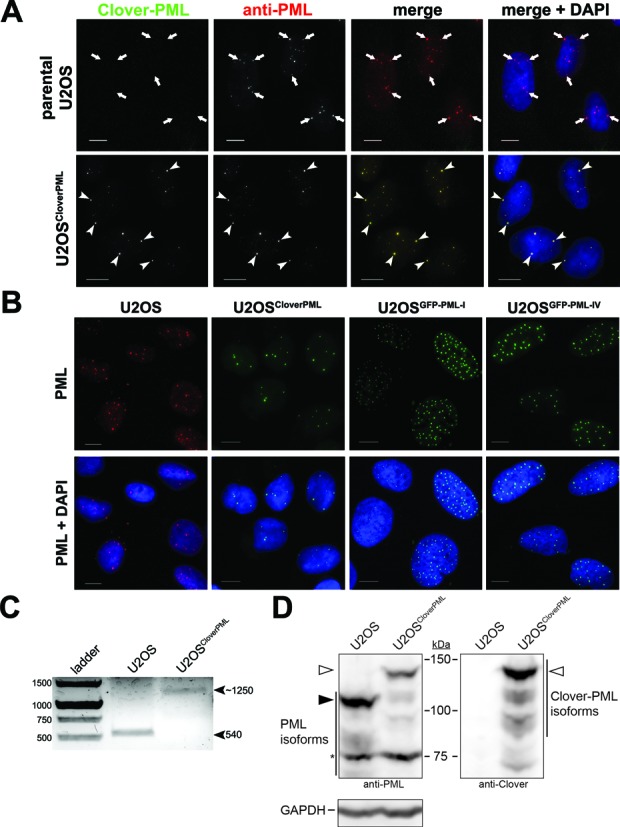
Expression of Clover-tagged PML in U2OS cells from the endogenous *PML* gene locus. (**A**) Parental U2OS and U2OS^Clover–PML^ cells were fixed and immunostained (anti-PML) to assess expression of Clover–PML and localization to PML NBs (merge + DAPI). (**B**) Comparison of representative PML NB intensity and number between parental U2OS cells (anti-PML immunostain), U2OS^Clover–PML^ cells (Clover fluorescence) and the CMV-promoter driven GFP-PML-I or GFP-PML-IV cells (GFP fluorescence) by fluorescence microscopy. For **A** and **B**: DNA was visualized with DAPI; Scale bar = 10 μm. (**C**) Genomic DNA was extracted from parental U2OS and U2OS^Clover–PML^ cells and the region around the *PML* translation start site in exon 1 was amplified by PCR and visualized by agarose gel electrophoresis and ethidium bromide staining. (**D**) Cell lysates from parental U2OS and U2OS^Clover–PML^ cells were analyzed by western blotting for Clover–PML (anti-GFP) and PML (anti-PML) as indicated. Solid arrow indicates untagged PML (isoforms 1 and 2). Open arrows indicate Clover-tagged PML isoforms 1 and 2. Asterisk (*) indicates a species that cross-reacts with the anti-PML antibodies.

We applied the CRISPR HDR gene targeting strategy described in Figure 4 to generate a U2OS cell line expressing PML from the endogenous locus with an N-terminal Clover tag inserted between the second and third residues of PML. U2OS cells were transfected with the two *PML* gRNAs, the Cas9^D10A^ nickase and the repair template. Five days after transfection cells were sorted by fluorescence activated cell sorting (FACS) to enrich for cells expressing Clover. Individual clones were isolated from this enriched population. To begin validating the selected U2OS^Clover–PML^ clone, we first examined whether the Clover fluorescent signal was associated with PML NBs. U2OS and U2OS^Clover–PML^ cells were immunostained for PML (Figure 5A). Clover signal co-localized with anti-PML immunostaining in the U2OS^Clover–PML^ clone, suggesting that at least one of the PML alleles was correctly edited to contain an in-frame N-terminal Clover tag.

We have previously used U2OS cells stably expressing GFP-tagged PML-I or PML-IV (29,43) to investigate PML biology. By comparison, the U2OS^Clover–PML^ cells most closely resembled the naive parental U2OS cells with respect to PML body size and number (Figure 5B) whereas the cells stably expressing GFP-PML-I or GFP-PML-IV cDNA from a CMV promoter had brighter anti-PML staining and higher PML body number (Figure 5B).

To confirm that the Clover sequence was correctly inserted into exon 1 of the *PML* gene, genomic DNA was extracted from the U2OS^Clover–PML^ and parental U2OS cells and amplified using PCR primers flanking the predicted Clover insertion site. A ∼540 bp fragment was amplified from parental U2OS cells, corresponding to the size predicted for the unedited genomic locus. A ∼1250 bp fragment that corresponds to the expected size for the correctly inserted Clover sequence was amplified from U2OS^Clover–PML^ cells (Figure 5C) and sequence verified (Supplementary Figure S4). Notably, we did not observe a ∼540 bp fragment in the PCR amplified from DNA extracted from the U2OS^Clover–PML^ cells, suggesting that all PML alleles contain the Clover insertion. The genome of U2OS cells is unstable and these cells contain many chromosomal abnormalities and duplications, with published karyotypes reporting between 3–4 copies of the *PML*-containing chromosome 15 (44,45).

The *PML* gene encodes seven major isoforms of PML which share a common N-terminus and vary in their C-termini (26–28). Inserting the Clover sequence at the beginning of the PML coding sequence should result in every PML isoform having an N-terminal Clover tag. To test whether multiple isoforms of Clover-tagged PML were expressed in the U2OS^Clover–PML^ cell line, protein extracted from the parental U2OS and U2OS^Clover–PML^ cell line was analyzed by western blotting (Figure 5D). In protein extracted from parental U2OS cells, a major species was detected with anti-PML antibodies migrating at around 110 kDa, consistent with the expected size of PML isoforms I and II. This species was not detected in the protein extracted from U2OS^Clover–PML^ cells, suggesting than untagged PML-I/II was not present. However, a prominent 130-kDa species was detected by both anti-PML and anti-Clover antibodies in protein extracted from U2OS^Clover–PML^ cells. This size is consistent with the predicted molecular weight for Clover-tagged PML-I/II. Several less abundant, lower molecular weight species of PML were also detected by the anti-PML antibodies in U2OS extracts, consistent with the approximate sizes expected for other PML isoforms. These species also appeared to exhibit a mobility shift corresponding to the size of the Clover tag in extracts of U2OS^Clover–PML^ cells, indicating that multiple isoforms had been tagged with Clover. Although several PML isoforms are expressed in the U2OS and U2OS^Clover–PML^ cells, the overall amount of PML protein is slightly lower in the U2OS^Clover–PML^ cells (Figure 5D).

**Figure 6.**
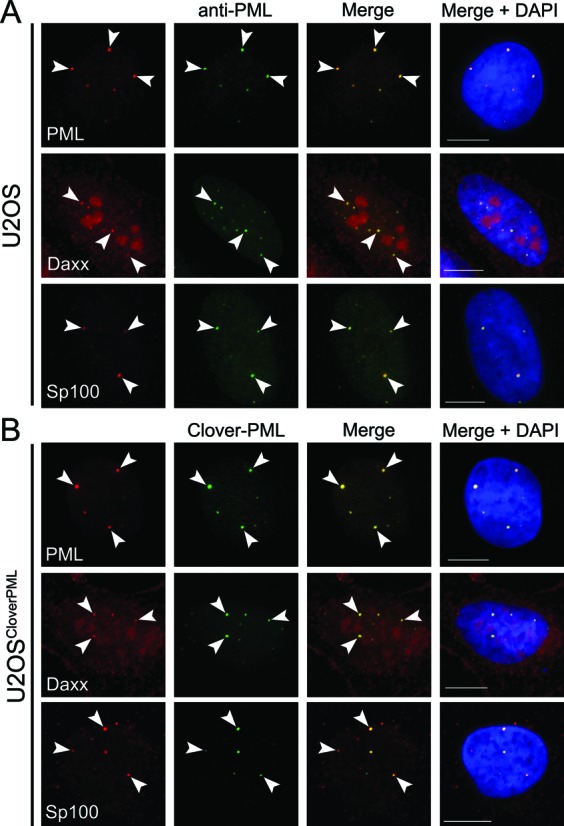
Clover-tagged PML forms PML nuclear bodies. Parental U2OS and U2OS^Clover–PML^ cells were fixed and immunostained for PML (rabbit anti-PML), Sp100 and Daxx as indicated (red). In addition, U2OS cells were immunostained with sheep anti-PML (green) while Clover–PML signal was detected in the U2OS^Clover–PML^ cells (green). Association of the indicated factors with PML nuclear bodies was assessed (merge panels) and examples of colocalization are indicated with arrows. DNA was visualized with DAPI. Scale bars = 10 μm.

We next wanted to investigate whether the PML NBs formed in the U2OS^Clover–PML^ cells contain known PML NB components, Sp100 and Daxx (42). Both Sp100 and Daxx were associated with PML nuclear bodies in parental U2OS cells (Figure 6A). Similar co-localization between Sp100 and Daxx with Clover–PML NBs was observed (Figure 6B), suggesting that the composition of PML NBs formed with Clover-tagged PML isoforms was not grossly perturbed.

**Figure 7.**
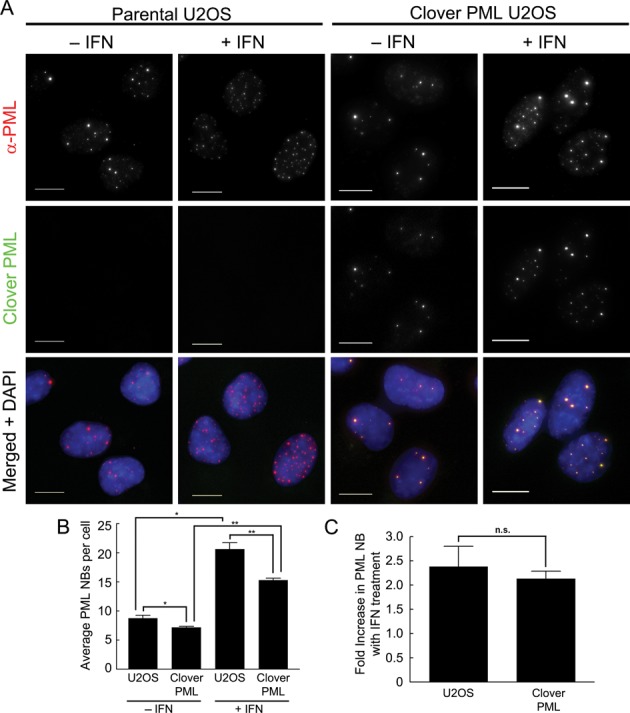
Interferon increases Clover–PML expression and PML NB formation. (**A**) U2OS and U2OS^Clover–PML^ cells were cultured with or without 1000 IU of interferon-γ (IFN) for 24 h and fluorescence microscopy of fixed samples was used to detect immunostained PML (anti-PML) and Clover–PML as indicated. DNA was visualized with DAPI. Scale bars = 10 μm. (**B**) The average number of PML NBs per cell was determined for U2OS and U2OS^Clover–PML^ cells +/− 1000 IU of IFN-γ for 24 h and immunostained (as described in **A**). (**C**) The fold-increase in the average number of PML nuclear bodies in interferon treated U2OS and U2OS^Clover–PML^ cells (from **B**) was calculated. For **B** and **C**, values represent the mean +/− standard error, n = 3. Student's t-test results are indicated with asterisks (* *= P* < 0.05, ** *= P* < 0.01, n.s. = not significant, *P* > 0.05).

Insertion of the Clover sequence at the 5′ end of the *PML* gene could affect normal regulation of the *PML* promoter. *PML* gene expression is induced in response to interferon (IFN) signaling, and the resulting increase in PML protein levels results in larger and more abundant PML NBs (46–48). We examined the interferon response in U2OS^Clover–PML^ cells. In response to 24 h treatment with 1000 IU of IFNγ, we observed a substantial increase in the number of PML NBs in the parental U2OS cells (Figure 7A). A similar increase in PML NBs was observed in the U2OS^Clover–PML^ cells (Figure 7A). We quantified the average number of PML NBs per cell for the parental and U2OS^Clover–PML^ cells with and without IFNγ treatment (Figure 7B). The U2OS^Clover–PML^ cells showed a small decrease in the average number of PML NBs per cell compared to the parental U2OS cells (7.19 versus 8.75, *P* = 0.038), consistent with the lower levels of the PML isoforms (Figure 5D). However, interferon treatment caused a significant increase in PML NBs for both the parental (*P* = 0.016) and U2OS^Clover–PML^ *(P* = 0.003) cells (Figure 7B). We calculated the average fold increase in PML NBs in response to IFN and found no significant (*P* = 0.463) difference between parental (2.38-fold) and U2OS^Clover–PML^ (2.13-fold) cells (Figure 7C). These results indicate that, although the U2OS^Clover–PML^ cells contain fewer PML bodies (82% of parental), the ability of the gene to respond to external stimuli (i.e. IFN) is unaffected by the lower body number, or the insertion of the Clover sequence at the PML locus.

## DISCUSSION

The ability to edit the mammalian genome with high efficiency will be critical in reaching the full potential of CRISPR/Cas9 and other genome engineering systems, for both scientific and clinical applications. Here we show that the small molecule RAD51 activator RS-1 increases the efficiency of Cas9-stimulated HDR between 3- to 6-fold, depending on the locus and transfection method. The HDR enhancement by RS-1 is compatible with wild-type Cas9, as shown in editing the *LMNA* locus, and for double-nicking Cas9, as shown for editing the *PML* locus. Further work will need to be performed to determine whether RS-1 also enhances HDR using other repair templates, such as single-stranded oligonucleotides. Using CRISPR/Cas9 for gene knock-in by HDR is currently much less efficient than generating deletions by NHEJ (3,18,19), eliciting the need to develop genome engineering protocols specifically optimized for HDR. Many design parameters can affect HDR efficiency, including the expression level of Cas9 and the gRNA(s), the amount of repair template, and homology arm length (49–52). Linearization of the repair template vector may promote HDR; in zebrafish, *in vivo* linearization of the repair template vector by addition of gRNA target sites flanking the homology arms increases HDR by almost 10-fold (53,54). We found that optimal ratio for Cas9/gRNA to homology donor plasmid for efficient HDR in our assay was 5:1 (Figure 2E) and this single parameter doubled the efficiency of HDR in our system. Optimizing both the gRNA target site and promoter that directs expression of the gRNA has also been shown to improve HDR efficiency (55,56). Activity of the HR repair pathway can depend on cell type and is limited to the S and G_2_ phases of the cell cycle (57). Synchronizing cells prior to transfection has been shown to be one method of increasing Cas9-induced HDR efficiency (58). Presumably, synchronization promotes the expression and stabilization of HR promoting singling pathways and proteins. Consistent with this concept, over-expression of wild type and hyper-recombination mutants of BRCA1 increased HDR in our assay by 2- to 3-fold (Figure 2D). Thus, manipulating DNA repair pathways through the addition of small molecules and transient expression of DNA damage response proteins, as we have done here, in order to favor HDR over NHEJ constitutes for a potent means of maximizing rates of HDR. Going forward, combining various approaches to enhance HDR will likely be necessary to fully optimize gene knock-in efficiency. These approaches may need to be optimized for individual cell lines and for each locus to be edited.

During preparation of this manuscript, another study reported a small molecule, L755507, that increased rates of Cas9-mediated HDR 2- to 3-fold in mouse cells (59). While we did not observe a significant increase in HDR efficiency by L755507 alone using our assay (*P* = 0.12), we observed a possible additive enhancement of combined treatment of RS-1 with L755507 over RS-1 alone; an effect that trended towards significance (*P* = 0.06) (Figure 3). In addition, it has been recently shown that treatment of embryonic stem cells with SCR7, a small molecule inhibitor of Ligase IV that inhibits NHEJ, could also enhance HDR (22,41). We also evaluated SCR7 using our knock-in HDR assay. Although SCR7 could enhance HDR as reported, it did so to a lesser degree than RS-1 in our assay. Furthermore, combined treatment with both SCR7 and RS-1 with or without L755507 did not further enhance HDR as compared to RS-1 alone (Figure 3).

We have also demonstrated the feasibility of generating knock-in cell lines encoding Clover-tagged nuclear domains. We characterized a U2OS cell line in which PML is tagged with Clover at the N-terminal region common to all PML isoforms. The Clover–PML isoforms appeared to form normal PML NBs that co-localized with the known PML body components Daxx and Sp100. The number of PML NBs in the U2OS^Clover–PML^ cell line was slightly lower than that in the parental line. This difference is consistent with the overall decreased levels of PML isoforms observed in U2OS^Clover–PML^ compared to parental U2OS cells. Insertion of the Clover sequence in the first exon of PML could slightly inhibit PML expression at the transcriptional or translational level. Alternatively, PML isoforms that are tagged with Clover may be slightly less stable than untagged PML. Regardless, PML NBs comprised of untagged or Clover-tagged PML both displayed a similar fold increase in number after cells were treated with interferon, suggesting that activation of the interferon response element in the PML promoter has not been perturbed by insertion of the Clover sequence.

Tagging PML at its endogenous locus will be particularly useful for live cell studies of PML dynamics. Our current knowledge of dynamics of the various PML isoforms was derived from experiments in which a single GFP-tagged PML isoform was over-expressed either in human cells having normal PML levels, or in a mouse PML knockout cell line (24,25,60,61). Since PML NBs are heterogeneous in their composition of individual PML isoforms, the ability to study PML isoforms without over-expressing PML or depleting endogenous PML may provide a more accurate representation of PML dynamics. Generating cell lines in which a gene is tagged at the endogenous locus to express a fluorescently tagged protein may also be useful in designing high-throughput screens, sensitive to changes in gene expression, and protein stability, for factors that affect levels of the fluorescently tagged protein.

## Supplementary Material

SUPPLEMENTARY DATA
