# Supplementary material for: Nuclear domain ‘knock-in’ screen for the evaluation and identification of small molecule enhancers of CRISPR-based genome editing
Source: Nucleic Acids Res. 2015 Oct 1;43(19):9379–92. doi: 10.1093/nar/gkv993 (PMC4627099; doi:10.1093/nar/gkv993)
Supplement: SUPPLEMENTARY DATA [file supp_43_19_9379__index.html]

Nuclear domain ‘knock-in’ screen for the evaluation and identification of small molecule enhancers of CRISPR-based genome editing — Nuclear domain ‘knock-in’ screen for the evaluation and identification of small molecule enhancers of CRISPR-based genome editing — SUPPLEMENTARY DATA 

# Nuclear domain ‘knock-in’ screen for the evaluation and identification of small molecule enhancers of CRISPR-based genome editing

## SUPPLEMENTARY DATA

- SUPPLEMENTARY DATA
