# Supplementary material for: Nuclear domain ‘knock-in’ screen for the evaluation and identification of small molecule enhancers of CRISPR-based genome editing
Source: Nucleic Acids Res. 2015 Oct 1;43(19):9379–92. doi: 10.1093/nar/gkv993 (PMC4627099; doi:10.1093/nar/gkv993)
Supplement: SUPPLEMENTARY DATA [file supp_gkv993_nar-00621-h-2015-File009.pdf]

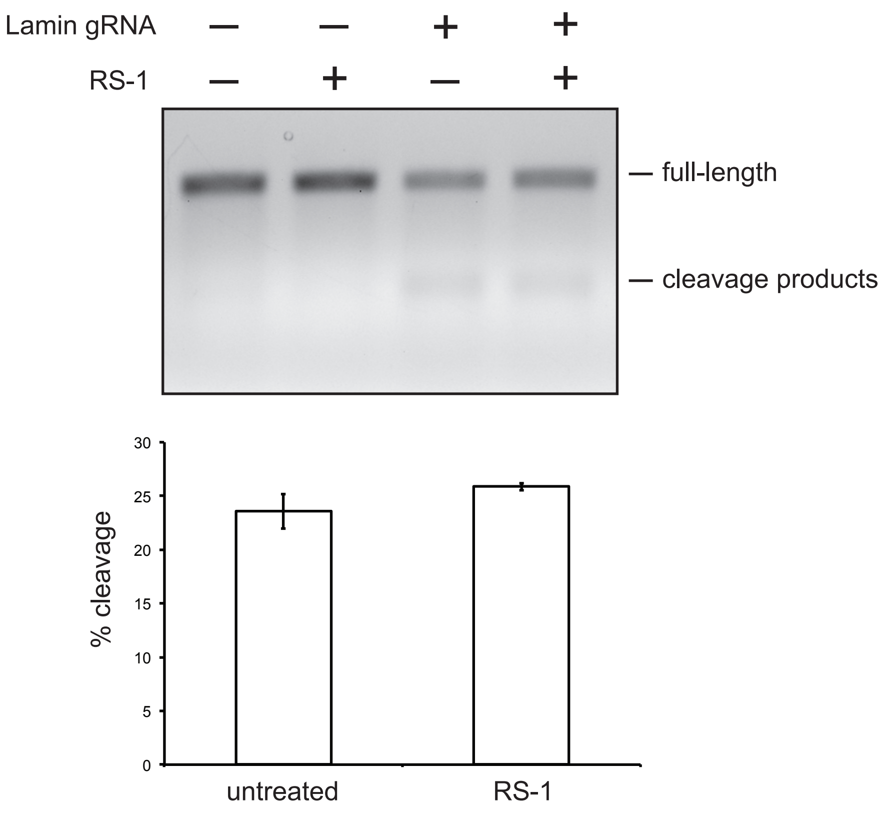

Supplementary Figure 1. Surveyor assay for measuring erroneous DNA repair at the LMNA locus. HEK293A cells transfected with pX330-LMNAgRNA1 were cultured for 3 days, and the region of LMNA locus flanking the gRNA target site was amplified by PCR from genomic DNA and subjected to a Surveyor nuclease assay. Digested PCR products were analyzed by ethidium bromide agarose gel electrophoresis and intensity of the full-length and cleavage products quantified by densitometry using Quantity One software (BioRad).

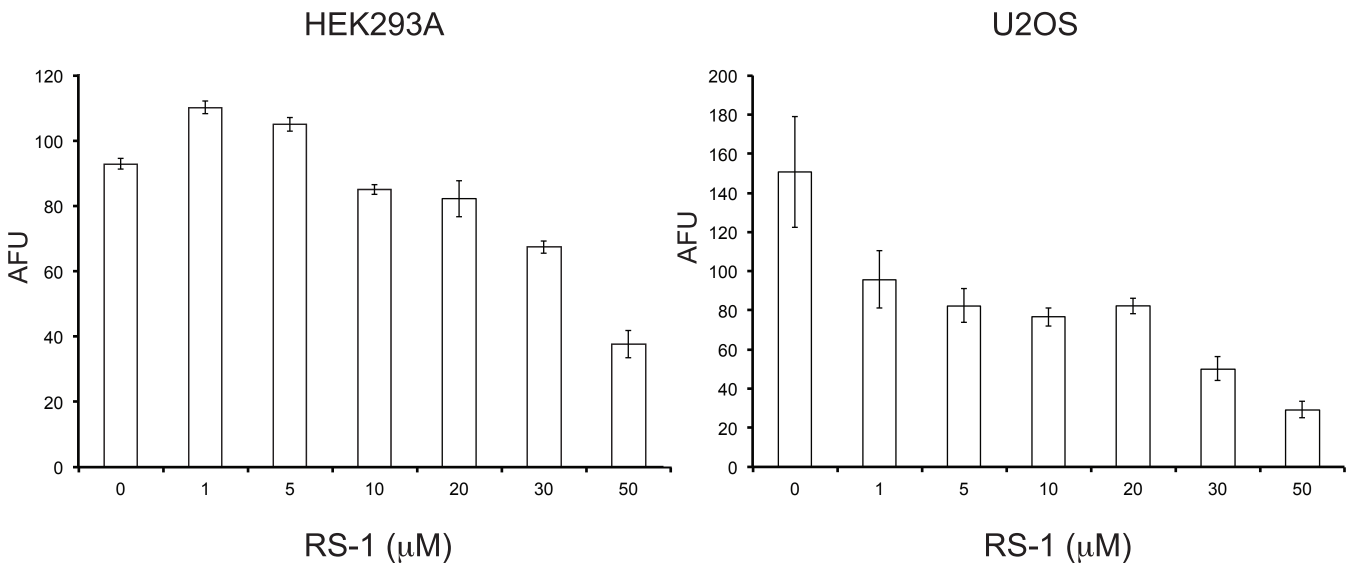

Supplementary Figure 2. Alamar blue assay for testing RS-1 cytotoxicity. Alamar blue fluorescence development (arbitrary fluorescence units, AFU) following culture of HEK293A or U2OS cells with the indicated concentrations of RS-1 for 72 hours.

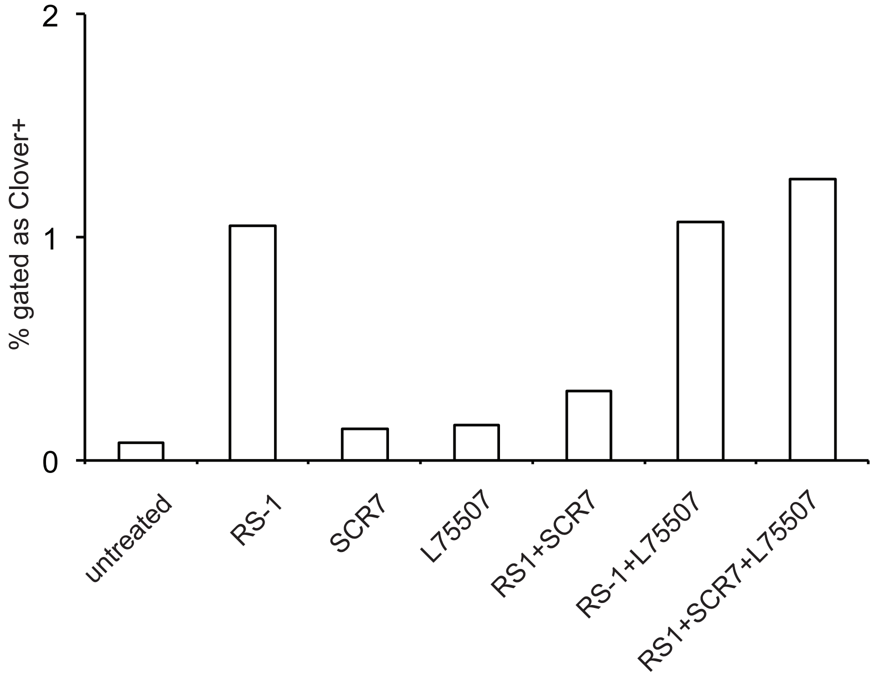

Supplementary Figure 3. Assessing contribution to fluorescence by RS-1, SCR7 and L755507. HEK293A were cultured in the absence of drug or in the presence of 10 μM RS-1, 1 μM SCR7, or 5 μM L755507 as indicated. Three days following transfection cells were fixed and analyzed by flow cytometry. Data represent the mean percentage of cells with sufficient fluorescence signal to be designated as “Clover-positive” counting at least 10 000 events.

Supplementary Figure 4. Clover-PML-U2OS Sequencing and Cloning Reference.

(A) Reference sequence for the region around the translation start site of PML exon 1. The coding sequence for PML is shown in bold. Guide RNAs targeting the PML locus are highlighted in yellow. The sequences used to generate the homology arms of the donor vector are shown in blue (5’ homology) and red (3’ homology). Grey highlighting indicates the region of the PML locus amplified from parental U2OS genomic DNA (Figure 4C).
(B) The Clover-PML repair template sequence consists of the 5’ (blue) and 3’ (red) homology arms which bracket the Clover sequence (green) which is inserted between the codons for amino acids 2 and 3 of PML. Grey highlighting indicates the region of the PML locus amplified from U2OS^{CloverPML} cells.

A. Reference sequence for 2340bp sequence around human PML gene Exon 1

Genomic Location: (hg19 chr15:74287154)

Legend:

Blue Text = Sequence used for 5’ homology repair template

Red Text = Sequence used for 3’ homology repair template

Yellow Highlights = sequence of guide RNAs

Underlined Text = Sequenced PCR amplicon from wild-type U2OS genomic DNA

Bold Text = coding sequence for PML exon 1

CCAGCACAGTGGTTGCGATGGTTTAAGTTTGAAGCCGGGGCGCTGACCCAGTGAGGTCAC
CTGGAACTGTGCCCCTTTCCCCTTACCAGCTGGGAGTCTGACATGCTTTTCCATTGGCGA
AGACCTAGCTGGCTCTCCCCTCACCCTCTTCATACCCGCTCTCCAGCTCTCCCCCTCCCT
CCTGCCCCCACCACCTCAGATCCACCAGCCTTTCCAGCTGTCACTCAAGGCATCATCCTG
CTCCCTCACCTCCAGTCACTGGCTTCCCAGGATCTCTGAATCAAGTCCCTCCTCTGCATC
CCACAGCCACTGTCCTAGACCAGGGCACCTGCTCCTAGGTTGTCAGAGTGACTGTCCCAT
CATGCACAGCTGATCGTGTTGTTCCCTGGTTCCAAATCCTTCAGCAACTTCCCATCACTC
ACAGAACTGGTTCAATCTCCTCAGCTAGGATCTTGTCCTAGCACACTCCTTGGGTATCAT
TTCTCATGAATTCCCTCTTAAATCTGAAAATGGCCAAAATTAGTTCCCAAACAGGCTTTT
GCAAGAGCTATCTTTTCTGTTTCAAGTGTCCCCCGTACCCCACCACCTACAACCCTAAAT
CCCTGGTGAATTTCCACTGCTTTTGCAGGTCCCAGCTGTGGGCTCTCCTTTCCAGAATGT
CTCCGTTTGCAGGCTTGCTCTTCCATTACATCTACGGCTCTTAGAAAGCAGGGGCTGCGT
GTGGCTCATCTTTGTACGCCCAGCTCCCGGCAGGACGTGGCACGAAGCAGTGCCAGTGTG
AACGGATGAATGGATCAAAGCCGGGAGCAGGGCTGCCCCCCTGCAGCTCTGCCCTACCTC
TCCCGCTTTACCGTAAGTCAGCGGTAGGTCTGCAGCTCTCCGCCTCTACCTCCTCCCCGC
TCTGGGCGTGTCTTTAAAACCCACAGTCGGCCTCTCTGCCCCCTAGAACCGCCCCCAGCT
TCTGTCTCACTTCCTCTCCAGAGGCGGGCCCTGAGCCGGCACCTCCCCTTTCGGACAGCT
CAAGGGACTCAGCCAACTGGCTCACGCCTCCCCTTCAGCTTCTCTTCACGCACTCCAAGA
TCTAAACCGAGAATCGAAACTAAGCTGGGGTCC**ATGGAGCCTGCACCCGCCCGATCTCCG**
AGGCCCCAGCAGGACCCCGCCCGGCCCCAGGAGCCCACCATGCCTCCCCCCGAGACCCCC
TCTGAAGGCCGCCAGCCCAGCCCCAGCCCCAGCCCTACAGAGGTACTATTGGGTTAGGGG
ATGATGGGGTTAAGCTTTGTTGGTTTGCTGTGGTGGGGAGAGGCGGGAAGAGAGGGTCTA
ACGGAGGATTTGGTCAAGTACCCTAGAGAGTGACACAAAGCGGGAAGTCCAGACACCAGG
GTCCTGACCGTCTCGGGTGGGGCAGGGAAGGGAGGGTAGGATAGAGTAGAAAAGAGGACA
CGGAGGAGTTGGGGCGGCCTCGCTGGGCTGCGGTTTCTCCACTGAGCAGTTGGGCAAGGT
GAGAAGGGTCAGTGGCCTCCGGGCCTGGGCCCTTCCGCCCACCCTCGAGCCCTGCCTCAA
CTTTGCCTCAGATGCAGGACTTCAGATTAGGGAGGATGGAGGTAGTACCCCTGTTGCGCT
GGCCTGGAGCCAGGGGCATGTCCCAGGCACGGCAAAACTAAAACCAACTTCCCAGATCCG
AGGTGAGAAACTGGCTCAGACTGAAGAGGTATCTTTGCCAAGGCCTCCCAGCTCATGTGG
TTTCTGCTTAAGGAAGCCTCCCCAACGAACCCTTCTCTTGCCACACCTTTCCTGCCCACC
TCCCACCTCCCCCGACAAAGGAACTACTTGGGTTTCTTGCTCTGCTGCCTTTCAGGCCCT
TTTACTCCCCTTCATGAAAGTACAGAGGACACCGTATTACAGTAACTTTTATAAACATTA
TTACAACTAAGAATAACATTACTTAACAATACTAGGTAACATTTATGAGCACTTCAAATG
TGCCAGGTACTGTATTAAGCACTTTGGTTTTTTTTTGTTGTTGTTTGTTTGTTTTGCTTT
GTTTTTTGTTTGTTTGTTTATCTGTTTGTTTTTGAGACGGAGTCTCACACTGTCGCCCAG
GCTGGAGTGCAATGGTGCGATCTCGGCTCACTGCAACCTCCTCCTTCCGGGTTCACGCCA
TTCTCCTGCCTCAGCCTCCCGAGTAGCTGGGATTACAGGCCACGCCACCATACCTGGCTA
TTTTTTATATTTTTAGTAGAGATGGGGTTTCACTATGTTGGCCAGATTGGTCTCTAACTC
CTGACCTCGTGATCCACCCACCTCGGCCTCCCAAAGTGCTGGGATTACAGGCATGAGCCA

B. Donor vector sequence for Clover-PML

Legend:

Blue Text = Sequence used for 5’ homology repair template

Red Text = Sequence used for 3’ homology repair template

Green Text = Sequence for Clover

Underlined Text = Sequenced PCR amplicon from clover-PML U2OS genomic DNA

Bold Text = coding sequence for PML exon 1 and clover

AAATTAGTTCCCAAACAGGCTTTTGCAAGAGCTATCTTTTCTGTTTCAAGTGTCCCCCGT
ACCCCACCACCTACAACCCTAAATCCCTGGTGAATTTCCACTGCTTTTGCAGGTCCCAGC
TGTGGGCTCTCCTTTCCAGAATGTCTCCGTTTGCAGGCTTGCTCTTCCATTACATCTACG
GCTCTTAGAAAGCAGGGGCTGCGTGTGGCTCATCTTTGTACGCCCAGCTCCCGGCAGGAC
GTGGCACGAAGCAGTGCCAGTGTGAACGGATGAATGGATCAAAGCCGGGAGCAGGGCTGC
CCCCCTGCAGCTCTGCCCTACCTCTCCCGCTTTACCGTAAGTCAGCGGTAGGTCTGCAGC
TCTCCGCCTCTACCTCCTCCCCGCTCTGGGCGTGTCTTTAAAACCCACAGTCGGCCTCTC
TGCCCCCTAGAACCGCCCCCAGCTTCTGTCTCACTTCCTCTCCAGAGGCGGGCCCTGAGC
CGGCACCTCCCCTTTCGGACAGCTCAAGGGACTCAGCCAACTGGCTCACGCCTCCCCTTC
AGCTTCTCTTCACGCACTCCAAGATCTAAACCGAGAATCGAAACTAAGCTGGGGTCC**ATG**
GAGGTGAGCAAGGGCGAGGAGCTGTTCACCGGGGTGGTGCCCATCCTGGTCGAGCTGGAC
GGCGACGTAAACGGCCACAAGTTCAGCGTCCGCGGCGAGGGCGAGGGCGATGCCACCAAC
GGCAAGCTGACCCTGAAGTTCATCTGCACCACCGGCAAGCTGCCCGTGCCCTGGCCCACC
CTCGTGACCACCTTCGGCTACGGCGTGGCCTGCTTCAGCCGCTACCCCGACCACATGAAG
CAGCACGACTTCTTCAAGTCCGCCATGCCCGAAGGCTACGTCCAGGAGCGCACCATCTCT
TTCAAGGACGACGGTACCTACAAGACCCGCGCCGAGGTGAAGTTCGAGGGCGACACCCTG
GTGAACCGCATCGAGCTGAAGGGCATCGACTTCAAGGAGGACGGCAACATCCTGGGGCAC
AAGCTGGAGTACAACTTCAACAGCCACAACGTCTATATCACGGCCGACAAGCAGAAGAAC
GGCATCAAGGCTAACTTCAAGATCCGCCACAACGTTGAGGACGGCAGCGTGCAGCTCGCC
GACCACTACCAGCAGAACACCCCCATCGGCGACGGCCCCGTGCTGCTGCCCGACAACCAC
TACCTGAGCCATCAGTCCGCCCTGAGCAAAGACCCCAACGAGAAGCGCGATCACATGGTC
CTGCTGGAGTTCGTGACCGCCGCCGGGATTACACATGGCATGGACGAGCTGTACAAGCCT
GCACCCGCCCGATCTCCGAGGCCCCAGCAGGACCCCGCCCGGCCCCAGGAGCCCACCATG
CCTCCCCCCGAGACCCCCTCTGAAGGCCGCCAGCCCAGCCCCAGCCCCAGCCCTACAGAG
GTACTATTGGGTTAGGGGATGATGGGGTTAAGCTTTGTTGGTTTGCTGTGGTGGGGAGAG
GCGGGAAGAGAGGGTCTAACGGAGGATTTGGTCAAGTACCCTAGAGAGTGACACAAAGCG
GGAAGTCCAGACACCAGGGTCCTGACCGTCTCGGGTGGGGCAGGGAAGGGAGGGTAGGAT
AGAGTAGAAAAGAGGACACGGAGGAGTTGGGGCGGCCTCGCTGGGCTGCGGTTTCTCCAC
TGAGCAGTTGGGCAAGGTGAGAAGGGTCAGTGGCCTCCGGGCCTGGGCCCTTCCGCCCAC
CCTCGAGCCCTGCCTCAACTTTGCCTCAGATGCAGGACTTCAGATTAGGGAGGATGGAGG
TAGTACCCCTGTTGCGCTGGCCTGGAGCCAGGGGCATGTCCCAGGCACGGCAAAACTAAA
ACCAACTTCCCAGATCCGAGGTGAGAAACTGGCTCAGACTGAAGAGGTATC

Supplementary Table 1. Plasmids used in this study.

Plasmid name	Description	Reference
pX330-U6-Chimeric_BB-CBh-hSpCas9	For expression of chimeric gRNA and wild-type Cas9	(2)
pX335-U6-Chimeric_BB-CBh-hSpCas9n(D10A)	For expression of chimeric gRNA and Cas9(D10A) nickase	(2)
pX330-LMNAgRNA1	gRNA targeting a sequence within the 5' region of the *LMNA* gene	This study
pX330-LMNAgRNA2	gRNA targeting a sequence within the 5' region of the *LMNA* gene	This study
pX335-PMLgRNA1	gRNA targeting a sequence within the 5' region of the *LMNA* gene	This study
pX335-PMLgRNA2	gRNA targeting a sequence within the 5' region of the *LMNA* gene	This study
pCR2.1-CloverPMLdonor	Clover sequence flanked by sequences homologous to the *PML* gene, for repair template	This study
pCR2.1-CloverLMNAdonor	Clover sequence flanked by sequences homologous to the *LMNA* gene, for repair template	This study
piRFP670-N1	For expression of far red fluorescent protein iRFP670	(32)
pHA-BRCA1	For expression of HA-tagged wild-type BRCA1	(33)
pHA-BRCA1(M1775R)	For expression of HA-tagged BRCA1(M1775R)	This study
pHA-BRCA1(K1702M)	For expression of HA-tagged BRCA1(K1702M)	This study

Supplementary Table 2. Primers for generating repair templates in this study

Primer name	Sequence (5' to 3')*	Amplifies
PML_5Hfwd	GTTCCCAAACAGGCTTTTGC	5' homology arm in PML
CloverPML_5Hrev	CTCCTCGCCCTTGCTCACCTCCATGGACCCCAGCTTAG	5' PML homology arm with Clover overhang
FLAGPML_5Hrev	CTTGTCATCGTCATCCTTGTAATCCTCCATGGACCCCAGCTTAG	5' homology arm in PML with FLAG overhang
PML_3Hrev	GATACCTCTTCAGTCTGAGC	3' homology arm in PML
CloverPML_3Hfwd	CATGGACGAGCTGTACAAGCCTGCACCCGCCCGATC	3' PML homology arm with Clover overhang
FLAGPML_3Hfwd	GATTACAAGGATGACGATGACAAGCCTGCACCCGCCCGATC	3' PML homology arm with FLAG overhang
CloverF	GTGAGCAAGGGCGAGGAG	Clover sequence
CloverR	CTTGTACAGCTCGTCCATG	Clover sequence
Lamin_5Hfwd	GGCAAGCTTGGAGCCGAC	5' LMNA homology arm
CloverLamin_5Hrev	CTCCTCGCCCTTGCTCACCTCCATGGCCGGCAGGTTG	5' LMNA homology arm with Clover overhang
Lamin_3Hrev	CAACTTGTCCCTGATACC	3' LMNA homology arm
CloverLamin_3Hfwd	CATGGACGAGCTGTACAAGACCCCGTCCCAGCGGCG	3' LMNA homology arm with Clover overhang

*5' primer extensions that do not match template are underlined
